# Characteristics of premanufacture CD8^+^ T cells determine CAR-T efficacy in patients with diffuse large B-cell lymphoma

**DOI:** 10.1038/s41392-023-01659-2

**Published:** 2023-10-25

**Authors:** Yao Wang, Chuan Tong, Yuting Lu, Zhiqiang Wu, Yelei Guo, Yang Liu, Jianshu Wei, Chunmeng Wang, Qingming Yang, Weidong Han

**Affiliations:** 1 Department of Bio-Therapeutic, The First Medical Center, Chinese PLA General Hospital, Beijing, China; 2 Changping Laboratory, Beijing, PR China; 3 National Clinical Research Center for Hematologic Diseases, The First Affiliated Hospital of Soochow University, Suzhou, China

**Keywords:** Cancer therapy, Immunotherapy

## Abstract

Although chimeric antigen receptor (CAR) T cells have become an important treatment option for patients with relapsed/refractory B-cell malignancies, more than 60% of patients with diffuse large B-cell lymphoma (DLBCL) treated with CAR-T cell therapies fail to achieve a durable response. To reveal changes in CAR-T cell therapy and identify response biomarkers, we conducted a retrospective analysis of pre-manufacture source T cells and CAR-T cell products and their association with outcome in 58 patients with r/rDLBCL who received tandem CD19/CD20 CAR-T cell therapy. We performed bulk RNA-Seq, single-cell RNA-Seq, and paired T cell receptor sequencing on CAR-T cell products and pre-manufacture T cells from DLBCL patients. We note that a CD8^+^ stem cell-like memory T cell population with a higher proportion and enhanced activating capacity of the CAR-T cell products was key to achieving durable clinical response. By analysing autologously-derived, pre-manufacture T cells, our data suggest that heterogeneity in the cellular and molecular features of pre-manufacture T cells contribute to the variation in efficacy after CAR-T cell therapy in DLBCL. The differences in anti-tumour efficacy of CAR-T cells among patients with different clinical outcomes appear to be due to the loss of CCR7 gene expression, coupled with increased expression of activation- and inhibitor-related genes in the CD8^+^ naïve-T cell populations among the apheresis T cells from patients with a poor molecular response. These findings significantly advance our understanding of the underlying molecular determinants of pre-manufacture T cell function.

## Introduction

DLBCL is a common aggressive haematologic malignancy,^1^ and ~60% of patients achieve complete remission (CR) after an initial anthracycline-based and rituximab-containing regimen.^2,3^ However, among patients with relapsed/refractory DLBCL (r/rDLBCL), the percentage of patients who achieve a 1-year survival rate is less than 30%.^4,5^ Although autologous anti-CD19 CAR-T cell therapies are highly effective in the treatment of large B-cell lymphomas, less than half of r/rDLBCL patients achieve remission for more than 1 year.^6,7^ In a retrospective study of 809 patients treated with commercially available anti-CD19 CAR-T cell therapy, the best overall response rate/complete response rate (ORR/CRR) was 66-80%/42-66%, the median overall survival (OS) following CAR-T cell infusion was 19.0 months and the median progression-free survival (PFS) was only 5.6 months.^8^ Identifying the factors that lead to treatment resistance and how to overcome them by designing innovative CAR-T cells has been a major focus of research in the field in recent years.

Loss or mutation of CD19 antigen expression is the common mechanism of CAR-T cell resistance and recurrence in B-cell malignancies.^9,10^ CAR-T cell designs that target two antigens simultaneously have been shown to reduce the rate of disease relapse due to antigen loss following infusion of CAR-T cells targeting a single antigen.^11–13^ Previously, we screened tandem CD19 and CD20 CAR-T cells (TanCAR7 T cells) and provided evidence of their efficacy in the treatment of recurrent/refractory non-Hodgkin lymphoma (r/rNHL).^14^ In one of our studies of 87 patients with r/rNHL treated with TanCAR7 T cells, the median PFS among DLBCL patients was 23.5 months.^15^ By contrast, in DLBCL, persistence and efficacy of tandem CAR-T cells seems beyond anti-CD19 CAR-T cells. However, more than a quarter of patients still do not achieve a clinical objective response, and ~30% of patients who have achieved a clinical response experience disease recurrence within a year. Antigen loss may explain some relapses, but not all relapsing patients have antigen-negative disease, suggesting that there may be other factors contributing to resistance.^16^

Some clinical data have shown that the level of detectable expansion and the duration of presence of CAR-T cells in vivo positively correlate with treatment efficacy and complete remission rates, and that patients who achieve rapid complete remission after cell infusion have a low relapse rate.^16,17^ Robust in vivo engraftment and expansion of CAR-T cells is a prerequisite for inducing anti-tumour efficacy.^16,18–20^ Evidence from some studies suggests that infused CAR-T cells lack the proportion of cells with a central memory phenotype and are less able to maintain long-term targeting of tumour cells in vivo.^19,21,22^ In addition, patients who receive infused CAR-T cells without memory cell populations tend to have higher relapse rates.^21^ Each CAR-T cell product is different because it is made from the patient’s specific T cells, and compromised apheresis-T cells result in a CAR-T cell product with reduced memory and activation function and susceptibility to premature exhaustion in vivo.^23–27^ Therefore, an important factor in the efficacy of CAR-T cell therapy is the difference between apheresis-T cells and CAR-T cells. However, this correlation has not been studied in the context of DLBCL patients receiving CAR-T cell therapy. Additional identification of factors that are associated with an increased risk of DLBCL progression following CAR-T cell therapy is required to enable early and potentially more effective therapeutic interventions.

Here we conducted a follow-up study of 58 DLBCL patients treated with CAR-T cell therapy, we analysed the killing of CD19/CD20-positive tumour cell line by infused CAR-T cells from all 56 evaluable patients in vitro, after excluding tumour-related factors, it was found that the anti-tumour function of CAR-T cells in resistant patients compared to the function of CAR-T cells in durable response (DR) patients. We then performed T cell subtype composition analysis in 56 evaluable patients, bulk RNA-seq analysis in 6 DR and 6 resistant patients and single-cell RNA-seq analysis in 6 patients including 3 DR and 3 resistant patients, and further analysed the correlation between the composition, function and gene expression of CAR-T cell products and apheresis cells and clinical efficacy to determine the characteristics associated with sustained clinical response. These data offer new insights into the cellular and molecular underpinnings of variability in the efficacy of CAR-T cell therapy among patients with DLBCL.

## Results

### The CAR-T cells in response patients have better anti-tumour ability and activation than those in resistant patients

**Fig. 1:**
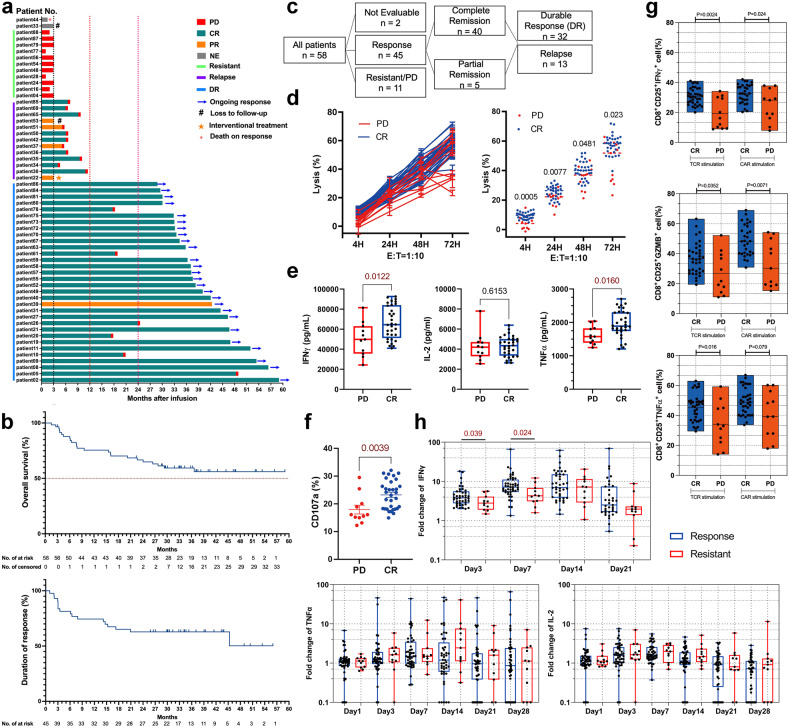
Evaluation of the clinical efficacy and anti-tumour function of tandem CAR-T cells in patients with r/rDLBCL. **a** Duration of response and survival after receiving the tandem CAR-T cell infusion. CR complete response, NE could not be evaluated, PD progressive disease, PR partial response. **b** Kaplan–Meier estimates of overall survival (up) and duration of response (down). **c** List of grouped in all 58 r/rDLBCL patients. **d** The CytoTox® 96 Non-Radioactive Cytotoxicity assay was employed to assess the cytotoxicity of CAR-T cells infused into Nalm6 cells at E:T ratio of 1:10. **e** Cytokine production by infused CAR-T cells co-cultured with Nalm6 cells at an E:T ratio of 1:1 for 24 h was measured using Luminex assays. **f** After coculturing CAR T-cells with Nalm6 cells at a 1:1 E:T ratio for 1 h, the expression of CD107a was observed. **g** Histogram plots of IFNγ, TNFα and GZMB expressed in infused CAR-T cells stimulated with small molecule mimics of TCR signalling. **d**–**g** CR patients *n* = 32; PD patients *n* = 11. **h** Serum cytokines, including TNFα, IL-2 and IFNγ, were measured in all evaluable patients, and their profiles are presented in this section. Response patients *n* = 45; Resistant patients *n* = 11. PD patients are resistant patients in this study

Between July 2017 and January 2020, 58 DLBCL patients were assigned to receive TanCAR7 T-cell therapy. As previously reported, among a total of 58 DLBCL patients, the best CRR was 71%.^15^ At the time of the study cut-off on May 10, 2022, 58% of the patients were still in remission, and 49% (95% CI, 34–64) showed no disease progression at 24 months after infusion (Fig. 1a). The median OS and duration response were not reached, and the estimated probability of survival was 67% (95% CI, 53–77) at 24 months (Fig. 1b). In this study, the response patients included patients who achieved CR or partial response (PR) after CAR-T cell infusion, and DR or relapse was defined based on whether those patients experienced disease recurrence within 1 year after cell treatment. Resistant patients who had progressive disease (PD) after CAR-T cell therapy. A total of 45 patients achieved responses; 32 out of these 45 patients were defined as DR; another 13 patients were defined as relapse; and 11 patients showed resistance to CAR-T cell therapy (Fig. 1c). We first analysed the relationship between factors of tumour heterogeneity and the efficacy of CAR-T cell therapy. Consistent with some data reported on NHL patients treated with anti-CD19 CAR-T cells,^7,20^ the best response to tandem CAR-T cell treatment was independent of the features of the patient’s previous treatment (number of lines of treatment, treatment resistance or relapse), tumour Ki67 expression and gene mutation (Supplementary Fig. S1).

To evaluate whether the clinical efficacy was due to inherent factors of CAR-T cell products, we used clinically manufactured CAR-T cells to kill human leukaemia cell line over time to assess the anti-tumour efficacy of patient CAR-T cells after excluding the effects of individual patient differences and tumour heterogeneity. The CAR-T cells obtained from CR patients were more effective at killing Naml6 tumour cells over 72 h than the CAR-T cells obtained from PD (resistant) patients (Fig. 1d). The CAR-T cells obtained from CR patients secreted significantly higher levels of TNFα and IFNγ than those obtained from PD patients (Fig. 1e). Analysis of CD107a expression on CAR-T cells confirmed this finding. After 1 h of coculture with Nalm6 cells, CD107a expression of CAR-T cells obtained from CR patients was higher than that of cells obtained from resistant patients (Fig. 1f). We next investigated the TCR- or CAR-specific functional properties of CAR-T cells in response to activation. Prior to analysis by intracellular flow cytometry, the infused CAR-T cells were stimulated for 4 h inducer of downstream TCR signalling (ionomycin) or Nalm6 cells. The CD8^+^CD25^+^ CAR-T cell populations in CR patients expressed higher levels of granzyme B (GZMB), IFNγ and TNFα than those in resistant patients after stimulation (Fig. 1g). In a retrospective analysis of the cytokine levels in 56 assessable patients after cell infused measured, significantly higher levels of serum IFNγ were observed at most time points within 6 weeks after cell infusion in patients who had resistance than in those who had a response (Fig. 1h). These data suggest that CAR-T cells from response patients have superior anti-tumour and activation capabilities compared to those from resistant patients before cell therapy.

### CD8^+^TSCM CAR-T cells play an important role in the response to CAR-T cell therapy

**Fig. 2:**
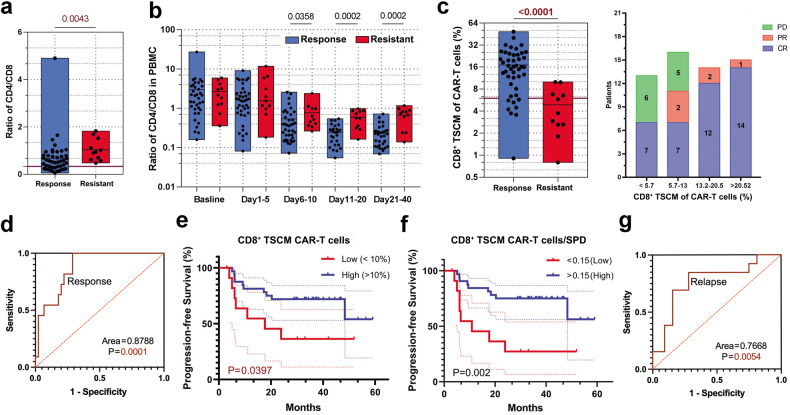
Correlation analysis of multiple factors of the CAR-T cell products with the treatment efficacy of CAR-T cells in DLBCL. **a** Analysis of the CD4/CD8 ratio of CAR-T cell products in the patients according to the response status. **b** Ratio of CD4^+^ and CD8^+^ CAR-T cells in blood measured in all patients at the indicated times. **a**, **b** Response patients *n* = 45; Resistant patients *n* = 11. **c** Analysis of CD8^+^ TSCM of infused CAR-T cells in patients by response status (left), quartile map of objective response among the 56 evaluable patients (right). **d** ROC curve for response prediction based on CD8^+^ TSCM population in CAR-T cell products consisting of frequency in the validation cohort (*n* = 56). **e**, **f** Kaplan–Meier analysis of PFS in response patients stratified by high versus low levels of the appointed index (*n* = 45). The dotted lines are the error bars. 0.15 is the quartile of the ratio of the CD8^+^ TSCM population in CAR-T cell products to the SPD (tumour burden). **g** ROC curve for recurrence prediction based on CD8^+^ TSCM population in CAR-T cell products normalised to tumour burden consisting of frequency in the validation cohort (*n* = 45)

Next, we sought to determine the key phenotypic characteristics of CAR-T cell products that were associated with clinical responses. The total number of infused CAR-T cells and CAR infection rate were not associated with the best response or durable response (Supplementary Fig. S2a). Several studies have reported that a higher proportion of terminal T cells expressed an exhaustion-related phenotype among CAR-T cell products, leads to the amplification and decreased memory ability of anti-CD19 CAR-T cells.^27–29^ In this study, it was observed that CAR-T cells obtained from resistant patients contained a slightly higher PD-1^+^TIM3^+^ cell population than those obtained from response patients, but there was no difference between DR and relapse patients (Supplementary Fig. S2b). The proportions of central memory T cells (TCM, CD45RA^−^CD62L^+^) and effector T cells (TEF, CD62L^−^) among the CAR-T products were not significantly different between response and resistant patients or between DR and relapsed patients (Supplementary Fig. S2c). The CD4/CD8 ratio of the CAR-T cell product in response patients was significantly lower than that in resistant patients (Fig. 2a). Correspondingly, CAR-T cell expansion in vivo was dominated by CD8^+^ T cells in response patients. This expansion started at 5 days after cell infusion and lasted for 3-4 weeks (Fig. 2b). Some studies of CD19-CAR-T cell therapy, patients who showed the best clinical outcome also had increased frequency of memory T cells in their CAR-T cell product.^20,27^ In our study, in CAR-T cell products of the all 56 assessable DLBCL patients, the proportion of CD8^+^ TSCM (stem cell-like memory T cells,^19,30,31^ CD45RA^+^CD62L^+^) was significantly higher in response patients than in resistant patients (*p* < 0.0001), and patients in the high quartile showed a response rate of 100% (Fig. 2c). CD8^+^ TSCM population successfully discriminated objective response and resistant patients based on the area under the areas under the ROC curve (Fig. 2d). Then, we assessed PFS using Kaplan–Meier analysis of 45 patients with achieved an objective response divided into two groups based on high versus low level of CD8^+^ TSCM population in CAR-T cell products. Patients with higher levels of CD8^+^ TSCM population had slightly better PFS (Fig. 2e). In patients with a large tumour burden, those with a high level of CD8^+^ TSCM content had a longer PFS (Fig. 2f). Notably, the number of CD8^+^ TSCM in CAR-T cell products significantly discriminated between durable remission and relapse (Fig. 2g). These data suggest that CD8-positive TSCM population among the CAR-T cell products was most significantly associated with an objective and durable response in r/rDLBCL patients treated with CAR-T cell therapy, especially in patients with a heavy tumour burden. The lack of CD8^+^ TSCM cells among the infused CAR-T cells may be a major factor related to primary treatment resistance.

**Fig. 3:**
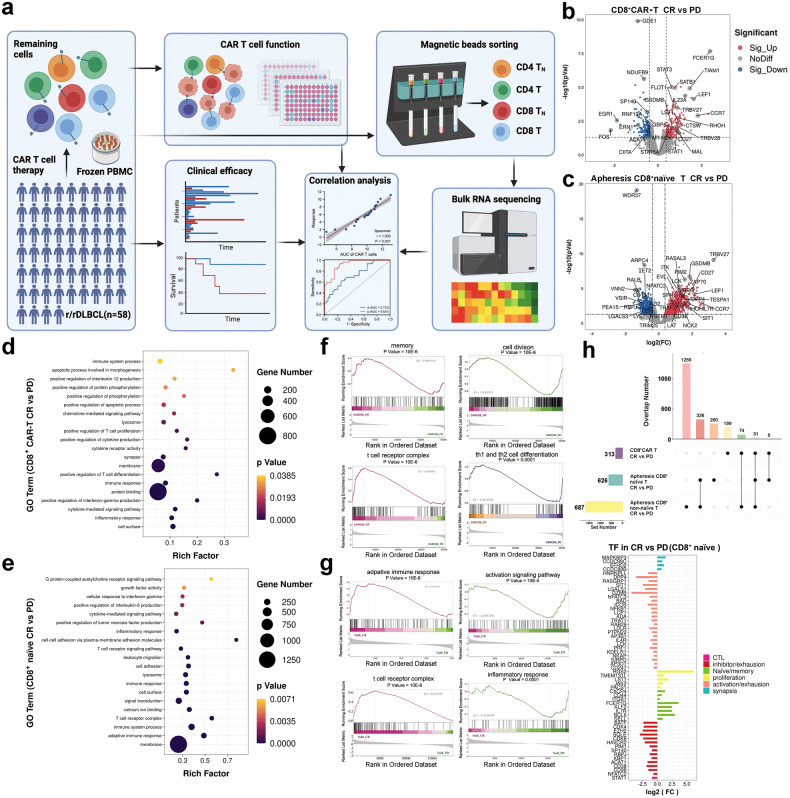
Bulk RNA-seq analysis of infused CD8^+^ CAR-T cells and premanufacture apheresis CD8^+^ naïve T cells. **a** Schematic of validation Bulk RNA-sequencing experimental design. Comparing resistant patients with DR patients, the volcano plot shows fold changes in gene expression in infused CD8^+^ CAR-T cells (**b**) apheresis CD8^+^ naïve T cells (**c**). Genes that have been upregulated are displayed in the colour red, while genes that have been downregulated are displayed in the colour blue. Genes that have not shown a statistically significant difference in expression are displayed in the colour grey. The log2 of the tag counts represents the values. The *p* value was reported as less than 0.05, indicating statistical significance. GO functional clustering of genes that were regulated in infused CD8^+^ CAR-T cells (**d**) apheresis CD8^+^ naïve T cells (**e**) of DR patients versus resistant/PD patients. Representative GSEA enrichment plot in infused CD8^+^ CAR-T cells (**f**) apheresis CD8^+^ naïve T cells (**g**) of DR patients versus resistant patients. **h** The Venn diagram illustrates the intersection of various TF genes (*p* < 0.05) among infused CD8^+^ CAR-T cells, apheresis CD8^+^ naïve T cells, and apheresis CD8^+^ non-naïve T cells of CR and PD patients. down: TFs in CD8^+^ naïve T cells significantly differentially expressed in CR versus PD patients

To determine whether durable remission with CAR-T cell therapy is the result of superior numbers of CD8^+^ CAR-T cells, we performed bulk RNA-seq analysis of CD8^+^ CAR-T cell products from DR and resistant patients (Fig. 3a). The sequencing samples were obtained from 6 resistant patients who showed significantly reduced killing efficacy in the previous efficacy experiment (Fig. 1d) and 6 DR patients who had a CR of over 24 months and disease characteristics similar to those of 6 resistant patients (Supplementary Fig. S3). There were differences in gene expression among the CD8^+^ CAR-T cell product between response and resistant patients (Fig. 3b and Supplementary Fig. S4a). Similar to the above phenotypic results, a majority of memory-related genes, such as CD27, TCF7, CCR7, LEF1, BACH2, SELL and CAPG, were significantly more highly expressed in CD8^+^ CAR-T cells in response patients than in resistant patients; moreover, the expression of activation- and exhaustion-related genes, such as GZMK, TBX21, EOMES, TOX, TIGIT, and SP140, were significantly downregulated (Fig. 3b and Supplementary Fig. S4b). Gene Ontology (GO) analysis showed that the activation of T cell related pathways, including activation, adhesion, proliferation and costimulation pathways, differed between response patients and resistant patients (Fig. 3d). The results of the gene set enrichment analysis (GSEA) showed that the expression of activation-related and cell proliferation-related genes was lower, while the expression of memory-related and complex-related genes was higher in the CD8^+^ CAR-T cells obtained from DR patients than in those obtained from resistant patients (Fig. 3f). Compared to CD8^+^ CAR-T cells in resistant patients, CD8^+^ CAR-T cells in DR patients were in a relatively resting state.

### Loss of CCR7 gene expression and increased expression of activation/inhibitor signature genes in infused CD8^+^ CAR-T cells correlate with resistance to CAR-T cell therapy

**Fig. 4:**
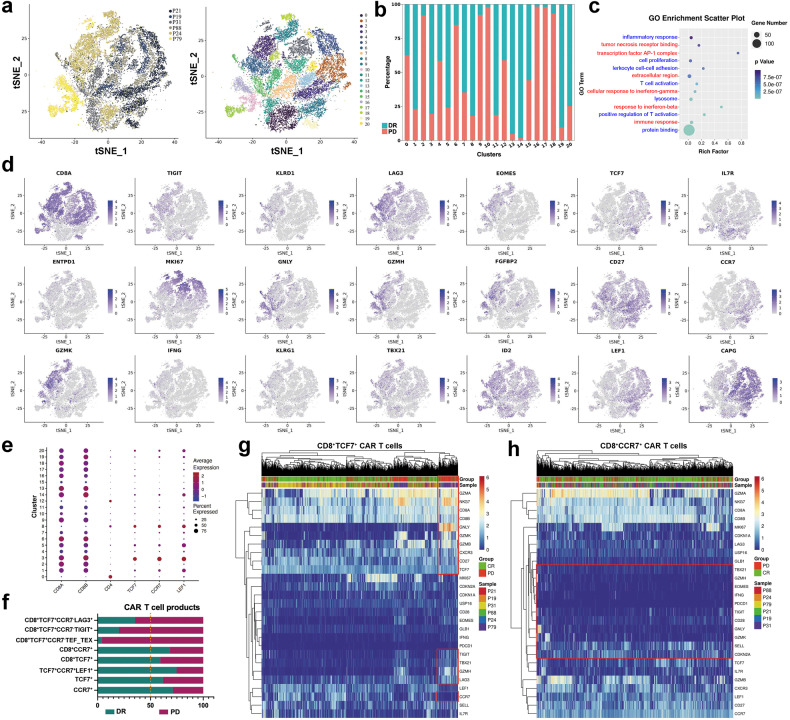
Single-cell RNA-seq analysis of infused CAR-T cell products. **a** Overview of all cells passing quality control for subsequent analysis in this study. In the tSNE plot, cells are colour coded on the left according to the corresponding patient origin (patient ID) and on the right according to the cluster number. **b** A bar graph showing the distribution of cells from DR and resistant patients among clusters. **c** The GO term dot plot illustrates the top ten differentially regulated pathways upon comparison of CAR-T cell infusions between DR patients and resistant patients. Differentially upregulated genes and pathways are in red; downregulated genes and pathways are in blue. **d** Expression of selected genes overlaid onto the tSNE plot. **e** The expression of T cell memory-associated markers is shown for each cluster. The size of the bubbles is proportional to the percentage of cells within a cluster that express a specific gene, while the colour corresponds to the average expression level of that gene within each cluster. **f** Quantification of the proportion of cells with different gene expression characteristics in DR and resistant patients. The heatmap displays the genes that are differentially expressed between CD8^+^TCF7^+^ CAR-T cells (**g**) CD8^+^CCR7^+^ CAR-T cells (**h**) obtained from patients with DR and those from patients with PD

In order to conduct a comprehensive characterisation of the outcome-associated CAR-T cell populations, we utilised 5′ scRNA-seq and paired T cell receptor sequencing to profile a group of infused CAR-T cells from three patients with DR and three patients with resistance. Single-cell data consisted of 28,348 cells from CAR-T products. Twenty-one subpopulations with distinct transcriptomic signatures were identified by data clustering and visualised using t-distributed stochastic neighbour embedding (tSNE) (Supplementary Fig. S5a, b). Clusters were defined based on genes related with cell state or function associated genes (Supplementary Fig. S5b), and Fig. 4a shows that all clusters contained cells from different samples. The relative proportion of cells from DR patients differed significantly from that from resistant patients (Fig. 4b), and we found that infused CAR-T cells were heterogeneous in terms of their T cell activation, cell proliferation, immune response, AP-1 complex, cell adhesion, lysosome, and response to interferon-gamma (Fig. 4c). The analysis of the cell cycle, conducted using 97 typical cell cycle markers^32^ revealed that CAR-T cells derived from patients with resistance or DR contained a similar subset of cells with analogous cell cycles (Supplementary Fig. S5c), suggesting that the functional differences in CAR-T cells are independent of the cell proliferation cycle. The cells in the C2, C6, C9–10, and C16–18 clusters expressed the highest level of resistant patient related genes (more than 80%) (Fig. 4b). The C6 cluster was defined as exhaustion/terminal CD8^+^ T cells and characterised by expression of LAG3, TIGIT, GZMB, GZMK, GZMA, GZMH, NKG7 and EOMES (Fig. 4d and Supplementary Fig. S5b). These signatures were highest in the C6 cluster, with progressively less expression in the C9, C15 and C16 clusters, and accompanied by a gradual increase in MKI67 expression.

We further analysed the above effector cell clusters that did not express memory-related genes including TCF7, LEF1, SELL and CCR7 (Fig. 4d), and found that the mean expression levels of the T cell activation-related transcription factors TBX21, EOMES and NKG7 gene were significantly higher in CAR-T cells from resistant patients than in those from DR patients, along with elevated expression of concomitant suppressors, including the CD38, LAG3, TIGIT, ADA, and ID2 genes, but the expression of the PDCD1 was low in the whole cell population (Supplementary Fig. S5d). Genes with senescence characteristics in CAR-T cells from resistant patients include CDKN1A, CDKN2A and USP16, although with elevated expression relative to DR patients, do not express CD39 and PDCD1, marking the above CCR7^−^TCF7^−^LEF1^−^ cells as predominantly effector cells, with cells from resistant patients in a relatively activated state (Supplementary Fig. S5d). Cells within the C1, C3, C5, C13, C14 and C19 clusters had the highest proportion of CD8^+^ T cells originating from patients who achieved a DR (more than 75%) (Fig. 4b, e).

TSCM cells, a rare subset of memory lymphocytes with stem cell-like properties defined by CD62L and TCF7, have an enhanced capacity for self-renewal and a multipotent ability to generate central memory, effector memory and effector T cells.^33–35^ In our study, TSCM cells were characterised by high expression of stem memory cell-like signatures, including TCF7, LEF1, SELL, CCR7, CD27, IL7R and TNFRSF6. The average expression levels of LEF1, CCR7 and TCF7 in TSCM cells in CAR-T cells of DR patients were higher than those of resistant patients (Supplementary Fig. S5e). We noted a significant increase in cells characterised by CCR7-positive expression in CD8^+^ CAR-T cells from DR patients (Fig. 4f). TSCM has a greater the abilities to self-renew and multipotency after TCR activation than TCM cells, mainly in the expression of CCR7.^34^ Interestingly, in CD8^+^TCF7^+^ CAR-T cells from resistant patients, loss of CCR7 expression was accompanied by expression of the above genes (Fig. 4g), but in CD8^+^CCR7^+^ CAR-T cells, the above gene expression was not significantly different between resistant and DR patients (Fig. 4h). It is suggested that reduced CCR7 expression in CD8^+^ memory CAR-T cells and increased expression of the activation/inhibitor genes LAG3, TIGIT, GZMB, GZMK, GZMA, GZMH, NKG7 and EOMES simultaneously in patients treated with CAR-T cells may be important gene expression features for treatment resistance.

The above results suggest that important transcriptional signatures of cells within CAR-T cell products are closely linked to clinical efficacy. There is a correlation between the efficacy of CAR-T cells and the mix of cells from DR and resistant patients in each cluster, as well as the relative proportion of cells from each state within each infused CAR-T cell.

### The composition and function of initially apheresis-T cells in patients treated with CAR-T cells are different before treatment

**Fig. 5:**
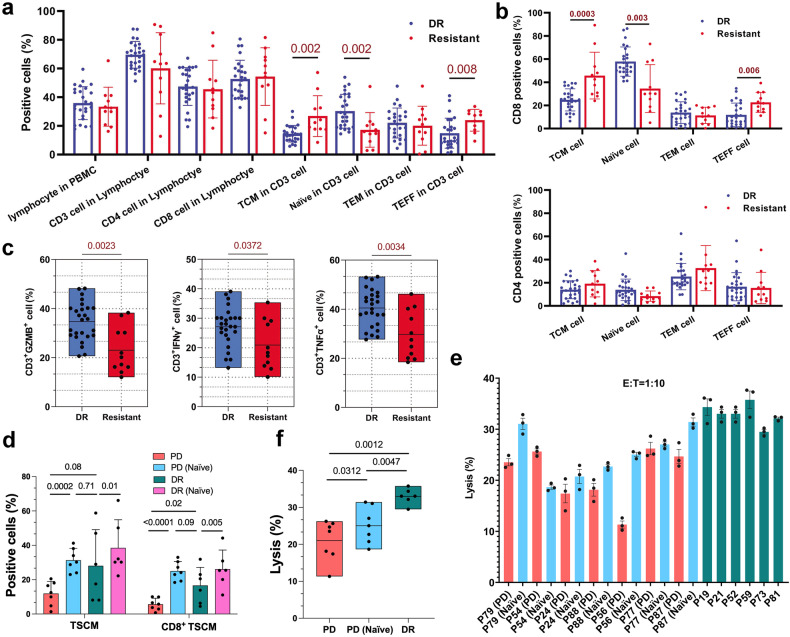
Analysis the composition and function of initial apheresis T cells. **a** Analysis of the cell population of apheresis T cells in DR and resistant patients. **b** Analysis of multiple indexes in CD8^+^ apheresis T cells (up) and CD4^+^ apheresis T cells (down) in DR and resistant patients. **c** Histogram plots of IFNγ, TNFα and GZMB expressed in apheresis T cells stimulated using small molecule mimics of TCR signalling. **a**–**c** DR patients, *n* = 26; PD patients *n* = 11. **d** The percentage of TSCM and CD8^+^ TSCM cell populations in CAR-T cells from different groups. **e** Cytotoxic activity of infused CAR-T cells and CAR-T cells cultured with naïve T cell of apheresis T cells with Nalm6 cells at an E:T ratio of 1:10. P-number indicated the unique number of patients in clinical trial (NCT03097770); P-number (PD) indicated CAR-T cultured with apheresis T cell from resistant patients; P-number (Naïve) indicated CAR-T cultured with naïve T cell from resistant patients. P-number (Naïve) indicated CAR-T cultured with naïve T cell from resistant patients. DR- number (Naïve) indicated CAR-T cultured with naïve T cell from DR patients. **f** Statistical analysis of cytotoxic activity of infused CAR-T cells and CAR-T cells cultured with naïve T cells. **d**–**f** DR patients *n* = 6; PD (resistant) patients *n* = 7

We hypothesised that infused CAR-T cell product characteristics may be a direct result of the differentiation state and function of patients’ initially T cells before cell therapy. The composition and function of the apheresis T cells in the 11 resistant patients and 26 long-time DR patients who had a durable response over 24 months were further analysed. We added the cell surface marker CCR7 to differentiate memory and effector cell populations. The levels of cell populations, including lymphocytes, T cell, CD4^+^ T cell, CD8^+^ T cell and TEM cell (CD45RA^−^CCR7^−^CD62L^−^), showed no difference between long-time DR and resistant patients, but the proportion of naïve T cell (CD45RA^+^CCR7^+^CD62L^+^) was significantly higher in DR patients than in resistant patients, while the proportions of TEFF cells (CD45RA^+^CCR7^−^CD62L^−^) and TCM cells (CD45RA^−^CCR7^+^CD62L^+^) were notably increased in resistant patients (Fig. 5a). These differences were mainly caused by the proportions of CD8^+^ T cells (Fig. 5b). The apheresis-T cells in DR patients expressed higher levels of GZMB, IFNγ and TNFα than those in resistant patients after stimulation of TCR signalling (Fig. 5c).

The result of the bulk RNA-seq analysis of the apheresis T cells is similar to the result of the CAR-T cells, gene expression differences were observed in CD8^+^ T cells from apheresis cells between response patients and resistant patients (Fig. 3c and Supplementary Fig. 4a). Importantly, DR patients and resistant patients had similar differences in genes in CD8^+^ naïve T cells and CD8^+^ CAR-T cells (Fig. 3c and Supplementary Fig. 4c), differential genes included memory-, proliferation-, synapses-, migration-, activation- and inhibitor-related TFs and genes (Fig. 3h). In addition, the genes involved in the immune process and the activation of interferon-gamma and tumour necrosis factor-alpha-mediated pathways were upregulated in CD8^+^ naïve T cells from DR patients compared to those from resistant patients (Fig. 3e). These differentially altered genes in the CD8^+^ naïve T cells obtained from DR patients were enriched in membrane composition, receptor-mediated signalling, T cell complex and calcium-ion channel (Fig. 3g). CD8^+^ non-naïve T cells also showed differential gene expression between response patients and resistant patients, but most of these genes were involved in the TCR signalling pathway (Supplementary Fig. 4c).

These data show that the difference in anti-tumour efficacy between the CAR-T cell products was already present in the T cells from which they were originally derived.

### A CD8^+^ T cell memory signature in pre-manufacturing apheresis T cells associate with durable response to CAR-T cell therapy

**Fig. 6:**
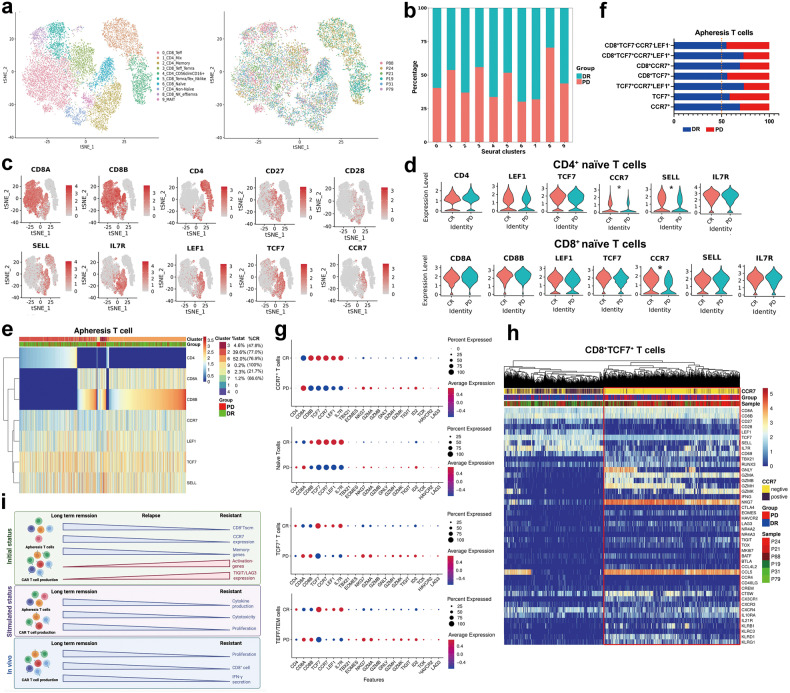
Single-cell RNA-seq analysis of apheresis T cells that share TCRs with CAR-T cell products. **a** On the right, cells are colour coded according to the corresponding patient origin (patient ID), while on the left, cells are colour coded according to the cluster number. The function of different clusters was defined according to differentially expressed genes. **b** A bar graph showing the distribution of cells from DR and PD patients among clusters. **c** Expression of selected genes overlaid onto the tSNE plot. **d** Violin plots showing selected gene expression in the CD4^+^ naïve (top) and CD8^+^ naïve (down) T cell populations. Stars indicate significance (*p* < 0.05). **e** The heatmap displays the genes that are differentially expressed between apheresis T cells obtained from patients with DR and those from patients with PD. **f** Quantification of the proportion of apheresis T cells with different gene expression characteristics in DR and PD patients. **g** The expression of T cell memory-associated genes and activation/inhibitory associated-markers are shown for different cells. The size of the bubbles is proportional to the percentage of cells within a cluster that express a specific gene, while the colour corresponds to the average expression level of that gene within each cluster. **h** Heatmap showing the differentially expressed genes between CD8^+^TCF7^+^ T cells of apheresis cells from DR and resistant patients. **i** Characterisation of functional/molecular differences of infused CAR-T cells and apheresis T cells between DR and resistant patients. Image (**i**) created with BioRender.com

To better identify and determine the characteristics and relationship between the CAR-T cell products and the pre-manufacturing T cells, here we selected only apheresis T cells that share TCRs with CAR-T cell products from 3 DR and 3 resistant patients and applied unsupervised clustering to these cells. Single-cell data consisted of 15,118 apheresis T cells, and ten subgroups with distinct transcriptomic signatures were identified by clustering (Fig. 6a). In the tSNE plot, apheresis T cells from resistant patients and DR patients overlapped each other and were not completely separated (Supplementary Fig. 6a, b), but the relative proportion of cells from DR patients was significantly higher in the CD4^+^ and CD8^+^ memory/naïve clusters (Fig. 6b, c). The expression of the CCR7, LEF1, AIF1, LTB and LST1 genes was significantly up-regulated in the cells of DR patients, while the CD69, GZMK, DUSP2, CXCR4, TIGIT and JUN genes were significantly up-regulated in the cells of resistant patients (Supplementary Fig. 6c). We first analysed the differences in the expression of CD8^+^ non-memory T cells in the two groups of patients. All CD8^+^ non-memory T cells exhibited high expression of NKG7, GZMA, GZMB, GNLY, GZMH and KLRD1, relatively high expression of TBX21 and EOMES, partial expression of GZMK, TIGIT, IFNG and TOX, but little expression of HAVCR2, PDCD1, TNFRSF9, CXCL13, MKI67 and ENTPD1 (Supplementary Fig. 6d). It was suggested that CD8^+^ non-memory T cells in both DR and resistant patients were dominated by low-growth effector cells and not terminal or exhaustion T cells. The relative proportions of cells in the non-memory T cell clusters were relatively similar in DR and resistant patients, but the average intensity of effector and inhibitor gene expression was higher in apheresis T cells from resistant patients than in those from DR patients (Fig. 6g).

We then focused on the expression of cells expressing memory-related genes in different patients. CD8^+^ naïve T cells and CD4^+^ naïve T cells were clustered into separate clusters (Fig. 6a), expressing both CCR7, TCF7, LEF1, SELL, CD27 and CD28 (Fig. 6c). Consistent with scRNA-seq of CAR-T cell products, the mean expression intensity of CCR7 was higher in CD4^+^ and CD8^+^ naïve T cells from DR patients than from resistant patients, with no difference in the expression intensity of other memory-related genes (Fig. 6d). As shown, the relative proportions of CD8^+^ naïve and CD4^+^ naïve cells of DR patients were over 70% (Fig. 6e). Additionally, the relative proportions of TCF7^+^ naïve T and TCF7^+^ memory cells from resistant patients were similar to those from DR patients, but the characteristics of CCR7 expression were able to distinguish memory cells in resistant patients from those in DR patients, and CCR7^+^ naïve cells and memory cells of apheresis T cells were significantly higher in DR patients than those in resistant patients (Fig. 6f, g). Resistant patients had significantly higher relative numbers of CD8^+^TCF7^+^CCR7^−^ cells than DR patients while expressing activation-related genes, including GZMB, GZMA and IFNG (Fig. 6g, h). This result is consistent with the analysis of CAR-T products, suggesting that functional memory phenotype differences in patients’ CAR-T cells already exist in pre-manufacture T cells, and CCR7^+^ T cells in resistant patients exhibited reduced memory function and increased expression of the activation- and inhibitor-related genes TIGIT and LAG3.

It has been reported that pre-selection of naïve-T cells before manipulation represents a crucial step for enriching TSCM.^19,36,37^ We sought to improve the cellular composition and anti-tumour function of CAR-T cell products for resistant patients by using naïve T cells as source cells for CAR-T cell manufacturing. The number of TSCM cell population was both effectively increased by CAR-T cells cultured with naïve T cells obtained from resistant patients and DR patients (Fig. 5d). The efficacy of CAR-T cells cultured with naïve T cells in killing of tumour cell line was higher than that of infused CAR-T cells but still lower than that of infused CAR-T cells obtained from long-time DR patients (Fig. 5e, f). These data demonstrate that the difference in anti-tumour efficacy among the CAR-T cell products may be determined by both the number and function of the pre-manufacturing naïve T cells.

## Discussion

First, we found that CAR-T cells from resistant patients had relatively lower anti-tumour killing and cytokine and degranulation production capacities in vitro. We then sought to determine whether the anti-tumour differences in CAR-T cell products between resistant patients and response patients were due to T cell intrinsic properties or cellular composition. In our data, the TEM, TCM and TEFF content of CAR-T cell products did not differ between DR patients and relapse and resistant patients, and only the composition of the CD8^+^ TSCM subset was significantly higher in DR patients than in other patients. TSCM is programmed to promote self-renewal and repress terminal differentiation, and overcome T cell exhaustion in cancer therapy.^34,35,38,39^ Several studies investigating the mechanism underlying clinical responses in B-cell malignancies have shown that CAR-T cell products enriched for early memory lineage subsets resulted better disease control,^20,21,25,27,40,41^ a scRNA-seq data of large B-cell lymphoma treated with CD19-CAR-T showed a 3-fold higher frequency of CD8^+^ CAR-T with memory characteristics in CR patients compared to PR/PD patients.^27^ Our data further suggest that the CD8^+^ TSCM composition may be an important factor in the efficacy and durable responses in r/rDLBCL patients treated with CAR-T cells.

It is noteworthy that the proportion of CD8^+^ naïve T cell in the apheresis T cells was significantly higher in DR patients than in resistant patients. It is possible that the content and composition of CD8^+^ TSCM in CAR-T cell products is influenced by differences in apheresis naïve T cells. We then further confirmed that in addition to the numerical advantage of the CD8^+^ TSCM population in CAR-T cells from DR patients, the functional gene expression was also significantly different from that of resistant patients. CD8-positive CAR-T cells from DR patients exhibited high expression of memory-associated TFs and genes. Disease recurrence or resistance may be attributed to suppression of CAR-T cells during prolonged exposure to antigens.^42^ During the “resting phase”, without contact with tumour cells, CD8^+^ CAR-T cells from resistant patients showed higher expression of activation/inhibitor-related genes, and exhibited a premature activation and differentiation state compared to DR patients (Fig. 4), which may be one of the reasons why CAR-T cells from resistant patients quickly became dysfunctional after stimulation with tumour cells in vivo.

Next, we used scRNA-seq to further identify important molecules in CD8^+^ TSCM that influence of durable clinical response to CAR-T cell therapy in DLBCL patients. There is functional significant heterogeneity in CAR-T cell products from DR patients compared to those from resistant patients. The number of CAR-T cells expressing TCF7, LEF1, CCR7 and CD27 on CD8^+^ TSCM cells in the DR patients was significantly higher than that in the resistant patients, in addition, the relative proportions of terminally differentiated cell populations expressing ID2, LAG3 and TIGIT were significantly higher in CD8^+^ CAR-T cells from resistant patients. Reduced TCF7 expression in T cells has been shown to cause patients to resist immunotherapy.^43–45^ However, in our results, TCF7 expression showed a slight difference between CAR-T cell products from DR patients and resistant patients. But, the difference in CCR7 expression in memory cells from CR patients compared to memory cells from PD patients was highly significant. Interestingly, cells with loss of CCR7 expression in CD8^+^TCF7^+^ cells from resistant patients were observed to have increased expression of both activation-related genes and some inhibitory marker genes such as TIGIT and LAG3.

The results of reduced CD8^+^ naïve-T cell numbers and reduced activation capacity prior to CAR-T cell manufacturing in resistant patients led us to speculate that the reduced anti-tumour function of CAR-T cell products was already present in the initial-T cells, the source cells from which the CAR-T product was manufactured. To test this hypothesis, we further analysed the gene expression of apheresis T cells that share TCRs with CAR-T cell products. As presumed, CD8^+^ naïve T cells in apheresis cells from resistant patients showed exhibited an activated state compared to those from DR patients, accompanied by decreased expression of the CCR7 gene and increased expression of partial inhibitor-related genes. TSCM has a greater the abilities to self-renew and multipotency after TCR activation than TCM cells, mainly in the expression of CCR7.^34^ Phenotypic, bulk RNA-seq and scRNA-seq results consistently showed that the absence of CCR7 gene expression, together with increased TIGIT/LAG3 gene expression in initial CD8^+^ naïve T cells and CAR-T cell products from resistant patients. We tried to prepare tandem CAR-T cells from naïve T cells in apheresis cells to compensate for the lack of TSCM cells in CAR-T cell products. However, in vitro experiments showed that although the ability to dissolve tumour cells could be partially improved, the ability of CAR-T cells in resistant patients to lyse tumour cells was still lower than that in cells from DR patients. These results further confirmed that the activation function of CD8^+^ naïve-T cells in apheresis cells directly affects the anti-tumour function of CAR-T cell products. For patients with reduced function of CD8^+^ naïve-T cells, the efficacy of CAR-T therapy may be further enhanced by early application of pharmacological interventions.

In summary, the content and function of CD8^+^ TSCM in CAR-T cells are important factors for patients to achieve long-term remission, and the impairment of initial CD8^+^ naïve T cells may determine CAR-T cell treatment resistance (Fig. 6i). Although the response to CAR-T therapy remains complex to decipher, the CD8^+^ TSCM in CAR-T cells is one piece of the puzzle to reduce treatment resistance and increase responsiveness to CAR-T cell treatment, CAR-T cell function can be improved and CAR-T therapeutic resistance can be reduced by collecting better source T cells (e.g., T-cell collection at earlier stage of disease diagnosis, or cell screening) for CAR-T cell preparation.

## Methods

### Patient samples

Clinical samples were collected from DLBCL patients who participated in a clinical study (ClinicalTrials.gov number NCT03097770) of tandem CD19/CD20 CAR-T cell therapy, which received approval from Chinese PLA General Hospital Ethics Committee. As part of the clinical trial, which was approved by the Institutional Review Board of Chinese PLA General Hospital, patients gave their informed consent to allow the use of their blood samples and de-identified health information for research purposes. Safety and efficacy results were previously reported.^14,15^

### Manufacturing of CAR-T Cells

CAR-T cells cocultured with naïve T cells were generated as previously described.^14,15,46,47^ Briefly, naïve T cells were isolated from apheresis T of patients using the Human naïve T Cell Enrichment Kit (Miltenyi Biotec) according to the manufacturer’s instructions, then the naïve T cells were activated using Human T-Activator CD3/CD28 magnetic beads (Dynabeads, Invitrogen) and cultured in X-VIVO 15 medium (Lonza) with the addition of 300 U/ml recombinant human IL-2 (PeproTech). After 48 h, activated T cells were transduced with the CD19/CD20 lentivirus vector. After CAR gene transduction, the cells were cultured outside the body with the addition of IL-2 three times a week until the desired number of cells was reached. The activated beads were magnetically removed when harvested cells.

### Cell line generation and culture

The Nalm-6 cell line of B lymphocyte leukaemia was acquired from ATCC (Manassas, VA). Subsequently, Nalm-6 cells expressing firefly fluorescein were constructed as described previously.^48^ The culture medium used for Nalm-6 cells was RPMI-1640 (Gibco), and it was supplemented with 10% fetal bovine serum (HyClone), 2 mM L-glutamine (Gibco), and 100 U/ml penicillin/streptomycin (HyClone).

### Flow cytometry analysis

To perform CAR expression, CAR-T cell samples were treated with a biotin-SP-AffiniPure F(ab)’2 fragment-specific goat anti-mouse IgG antibody (Jackson ImmunoResearch). Subsequently, streptavidin-phycoerythrin (PE) or streptavidin-fluorescein isothiocyanate (FITC) were added during the incubation process.

Flow cytometry was performed on CAR-T cells or T cells using the following antibodies: CD3 (PerCP and allophycocyanin, APC), CD8 (PE or PerCP), CD4 (FITC), CD45RA (APC), CD62L (PE), PD-1 (APC), TIM3 (PE), LAG3 (FITC), CCR7 (APC), GZMB (APC), TNFα (APC) and IFNγ (PC5.5). Degranulation experiment, CAR-T cells were cocultured with Nalm6 cells in RPMI-1640 medium. During the assay, CD107a antibody was added, followed by incubation with a Golgi Plug protein transport inhibitor for 3 h.

All antibodies were purchased from BD Biosciences and used at the recommended concentration. Isotype control monoclonal antibodies were used in addition to the flow-through assay. Flow cytometry was performed on a Beckman DxFLEX instrument and data analysis was performed with FlowJo software (Version 10.0.7, FlowJo, Ashland, OR).

### In vitro functional assays

CAR-T cells were co-cultured with Nalm6 cells at an E:T ratio of 1:10 in 96-well plates. Next, 100 microlitres of a solution containing twice the concentration of D-luciferin (300 micrograms per millilitre) was added to each well. Signals were then recorded using Varioskan™ LUX (Thermo Fisher) after 3 to 5 min. Furthermore, the levels of cytokines were measured using enzyme-linked immunosorbent assay kit (ELISA kit; Novus Biologicals) and Luminex assay.

### Measurement of serum cytokine levels

Patient serum was collected at the indicated time points based on the clinical protocol and serum levels of cytokines (IL-2, TNFα and IFNγ) were determined by ELISA kit (Novus Biologicals) using the Luminex assay according to the manufacturer’s instructions.

### Isolation of CAR-T cells for RNA-sequencing analyses

The 500,000 purified CAR-T cells were acquired by sorting kit (magnetic beads, Miltenyi Biotec) in accordance with the manufacturer’s instructions from the TanCAR7 cell product of the designated patient. The sorted CAR-T cells were subjected to bulk RNA-seq and scRNA-seq.

### RNA-sequencing analysis

Each sequenced library’s read counts were adjusted using the edgeR package and a scaling factor before differential gene expression analysis. The statistical analysis software R/Bioconductor used the DEseq2 package version 1.16.1 to analyse the normalised RNA-seq data. The empirical Bayesian approach was used in generalised linear models to detect genes that were differentially expressed in a significant manner. A significant difference was considered when the *p* value < 0.05.

### Single-cell sequencing data processing

The sequencing results were demultiplexed and converted to FASTQ format using the Illumina bcl2fastq program (version 2.20). The Cell Ranger pipeline (https//support.10xgenomics.com/single-cell-geneexpression/software/pipelines/latest/what-is-cell-ranger, version 6.1.1) was used for demultiplexing, barcode processing, and single-cell 3’ gene counting. The scRNA-seq data were aligned to the Ensembl GRCh38/GRCm38 reference genome.10X Genomics Chromium Single-Cell 3’Solution was used to process a total of 110,000 single cells obtained from sorted CAR-T cells and apheresis T cells from six patients. Seurat (version 3.1.1) was used to load the Cell Ranger output to perform dimensional reduction, clustering and analysis of the scRNA-seq data. A total of 60,000 cells met quality control criteria. All genes expressed in fewer than three cells were excluded. The number of genes expressed per cell was considered low if greater than 500 and high if less than 5000. UMI counts below 500 were not considered, and gene expression derived from mitochondrial DNA was less than 25%.

### Statistical analysis

STATA version 15.0 was used for all statistical analyses. To determine the statistical significance between two unpaired sample groups, unpaired two-tailed Wilcoxon–Mann–Whitney *U* tests were employed. For categorical variables, precise techniques such as Clopper-Pearson 95% confidence intervals have been used. For forest plots, confidence intervals are presented for each group and for the comparison between the groups and the reference group. For time-to-event analyses of PFS and OS, the starting point was the infusion of CAR-T cells. A Kaplan–Meier method was used to calculate PFS and OS, and a log-rank test was used to compare them. An exact Wilcoxon rank-sum test was used to determine differences between two categories with respect to the continuous parameter. We calculated the area under the receiver operating characteristic (ROC) curve to evaluate the sensitivity of the multi-index in predicting response or relapse to TanCAR7 T cells.

### Supplementary information

Supplementary_Materials

## Data Availability

Bulk RNA-seq data have been deposited in Gene Expression Omnibus under accession no. GSE223655. Single-cell RNA-seq data have been deposited in Gene Expression Omnibus under accession no. GSE243325. All data in this article are available upon reasonable request from the corresponding authors.
