# Supplementary material for: Characteristics of premanufacture CD8+ T cells determine CAR-T efficacy in patients with diffuse large B-cell lymphoma
Source: Signal Transduct Target Ther. 2023 Oct 25;8:409. doi: 10.1038/s41392-023-01659-2 (PMC10598004; doi:10.1038/s41392-023-01659-2)
Supplement: Supplementary file 1 — Supplementary_Materials [file 41392_2023_1659_MOESM1_ESM.docx]

Supplementary Materials for

**Characteristics of premanufacture CD8^+^T cells determine CAR-T efficacy in patients with diffuse large B-cell lymphoma**

Yao Wang, Chuan Tong, Yuting Lu, Zhiqiang Wu, Yelei Guo, Yang Liu, Jianshu Wei, Chunmeng Wang, Qingming Yang, Weidong Han

Correspondence to: [wangyao_301@hotmail.com](mailto:wangyao_301@hotmail.com) or [hanwdrsw@163.com](mailto:hanwdrsw@163.com)

**This PDF file includes:**

Materials and Methods

Supplementary Figures. S1 to S6

**Supplementary Materials and Methods**

**Efficacy assessments**

The primary analysis of efficacy assessments will be based on the recommendations by the International Malignant Lymphomas Imaging Working Group (Cheson Response Criteria and The Lugano Classification 2014).

A patient evaluated to have a response of CR must show no evidence of bone marrow aspirate/biopsy by morphology or by immunohistochemistry (if the bone marrow was involved by lymphoma at baseline), and the spleen and liver must be normal in size with no lymphoma-related B-symptoms in addition to radiological CR.

• A patient will have a best overall disease response of partial response (PR) if there is a >50% decrease in the SPD of the six largest dominant nodes or nodal masses and no increase in the size of the other nodes, liver or spleen (and the patient would not qualify for CR).

• A patient will be evaluated as having PD if there is a >50% increase from nadir in the SPD of any previously identified abnormal node or if the appearance of any new lesion that is >1.5 cm by radiologic evaluation was observed.

• If a patient does not qualify for CR, PR, SD or PD, then their disease response will be not evaluated (NE).

The objective response rate (ORR) is defined as the proportion of patients with either CR or PR per the Lugano Classification (Cheson et al, 2014) as the overall disease response while on study.

The best ORR is defined as the incidence of CR and PR recorded from TanCAR7 T cell infusion until PD or the start of new antitumour therapy, whichever comes first.

**RNA sequencing data processing.**

The first step was to process the raw data in fastq format, resulting in the acquisition of clean data (referred to as clean reads). This was achieved by eliminating reads containing adapters, reads with poly-N sequences and low-quality reads from the raw data. FastQC performs the quality control of the raw fastq data. The reference genome index was constructed using Hisat2 v2.0.5 and the paired-end clean reads were aligned to the reference genome using Hisat2 v2.0.5.Trim Galore (https //github.com/FelixKrueger/TrimGalore) was used to trim the paired-end reads. The cleaned reads were aligned to the UCSC Human GRCh38/hg38 reference genome available at http://genome.ucsc.edu/.StringTie (v1.3.3b). The mapped reads from each sample were assembled. HTSeq (Hisat2 v2.0.5) counted the trimmed reads that had a mapping quality greater than 20.The FPKM for each gene was determined by dividing the number of reads mapped to the gene by the length of the gene.

**Single-cell sequencing.**

Using the manufacturer's instructions of the 10X Genomics Chromium Single-Cell 3'kit (V3), 10000 single cells were captured by loading single-cell suspensions onto 10x Chromium. According to the standard procedure, the subsequent steps of cDNA amplification and library construction were carried out. Libraries were sequenced by LC-Bio Technology Co. Ltd. (Hangzhou, China) on an Illumina NovaSeq 6000 sequencing system (paired-end multiplexing run, 150bp), achieving a minimum depth of 20,000 reads per cell.

**Supplementary Figures**


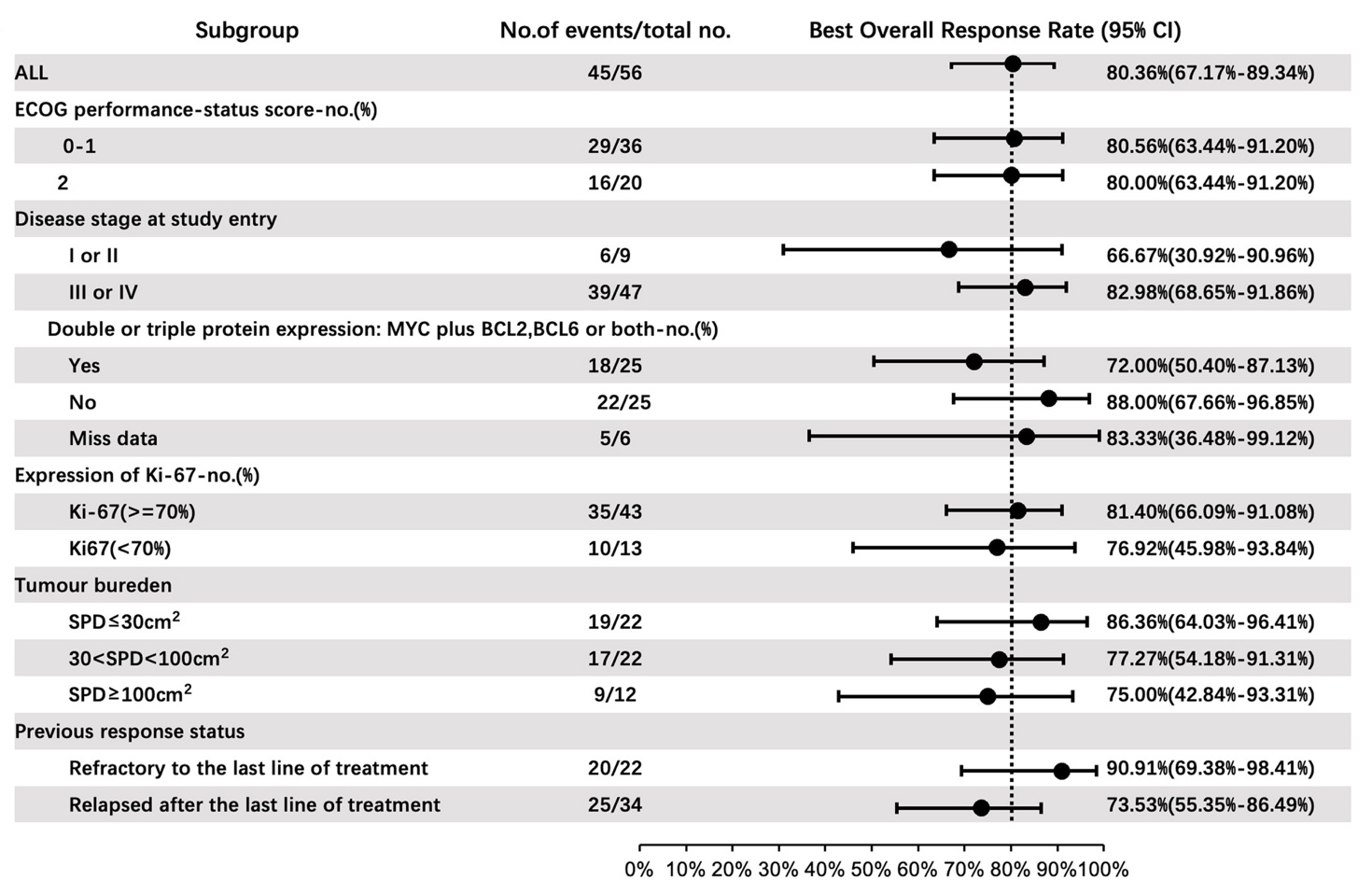


**Supplementary Figure. S1. Best overall response rate based on subgroup.** Best overall response rate based on subgroup. ECOG Eastern Cooperative Oncology Group, SPD sum of the product of the diameters.


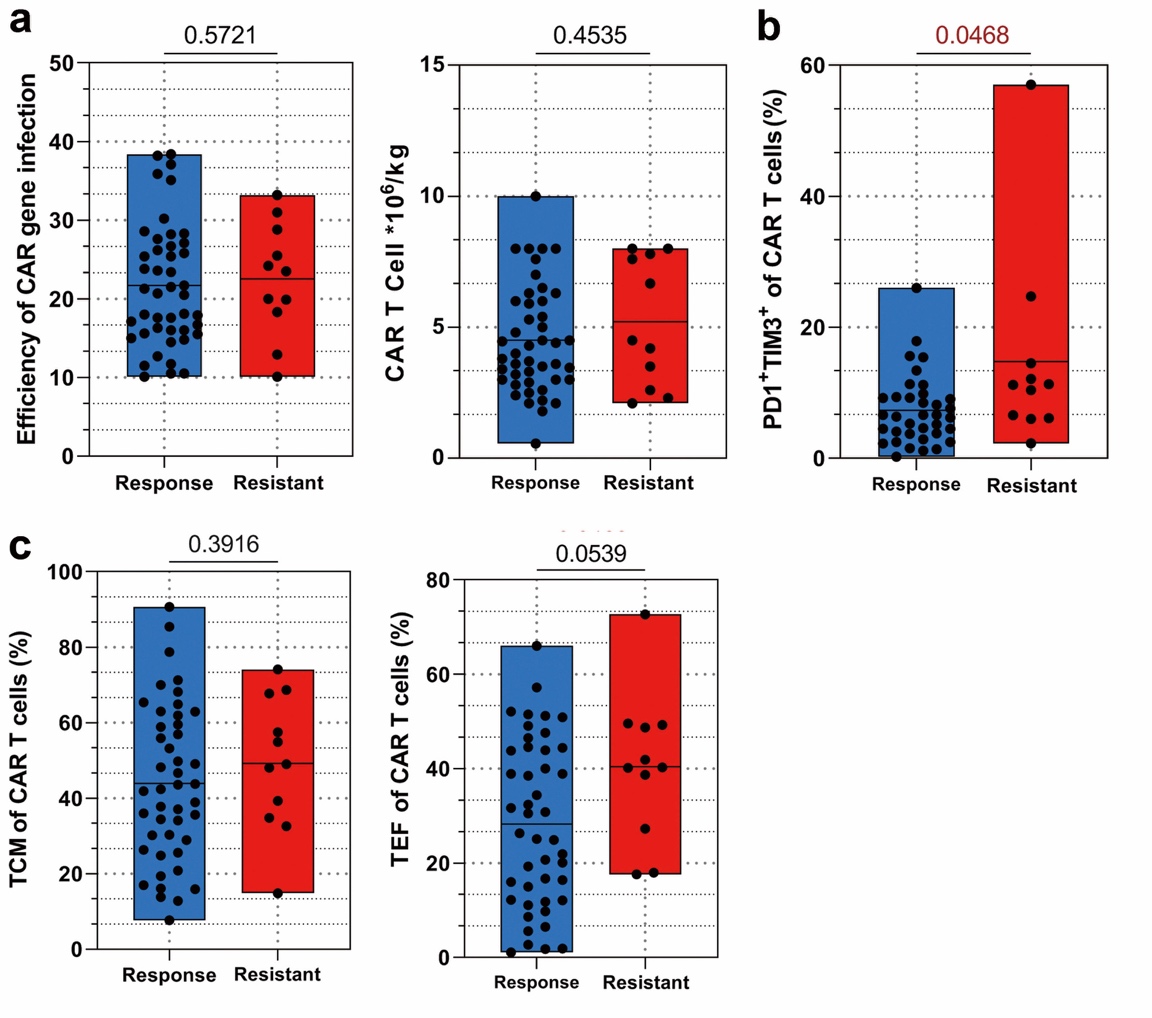


**Supplementary Figure. S2. Correlation between phenotype of CAR-T cell products and efficacy. a.** Analysis of efficiency of CAR gene infection of infused CAR-T cells **(left)** the total number of infused CAR-T cells of 56 evaluable patients **(right)** by response status. Analysis of PD1^+^TIM3^+^ cell population (**b**) TEF or TCM (**c**) cell population in infused CAR-T cell products of 56 evaluable patients by response status. TCM: central memory T cells, CD45RA^-^CD62L^+^; TEF: effector T cells, CD62L^-^**.**


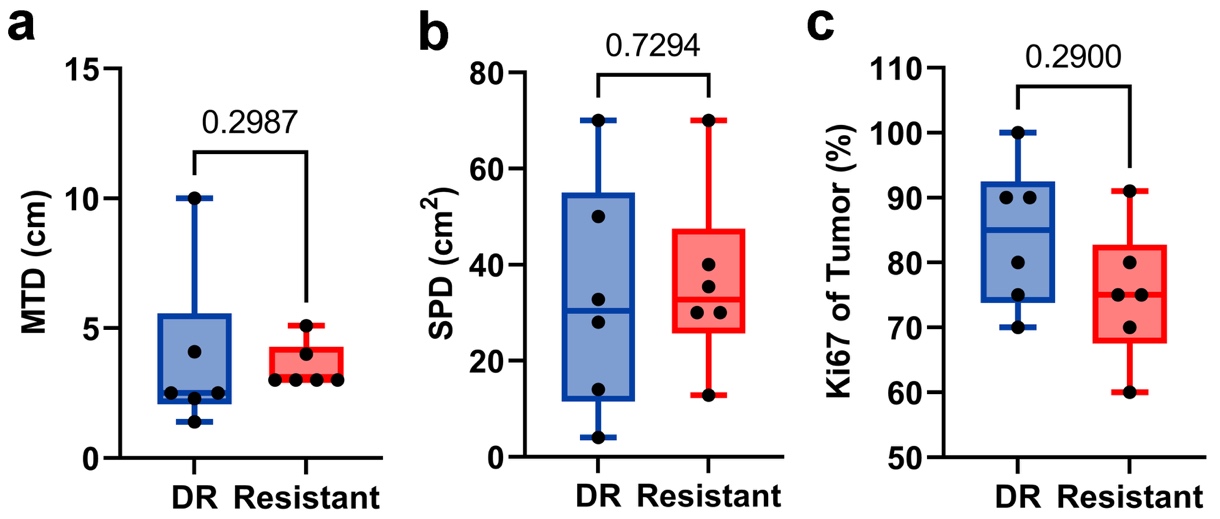


**Supplementary Figure. S3. Analysis of multiple clinical characteristics of patients whose samples were sequenced.** in MTD (**a**) SPD (**b**) and ki67 expression of tumour (**c**) in 6 DR patients who had a CR of over 24 months and 6 relapsed patients. MTD: maximum tumour diameter, SPD: the sum of the product of the diameters.


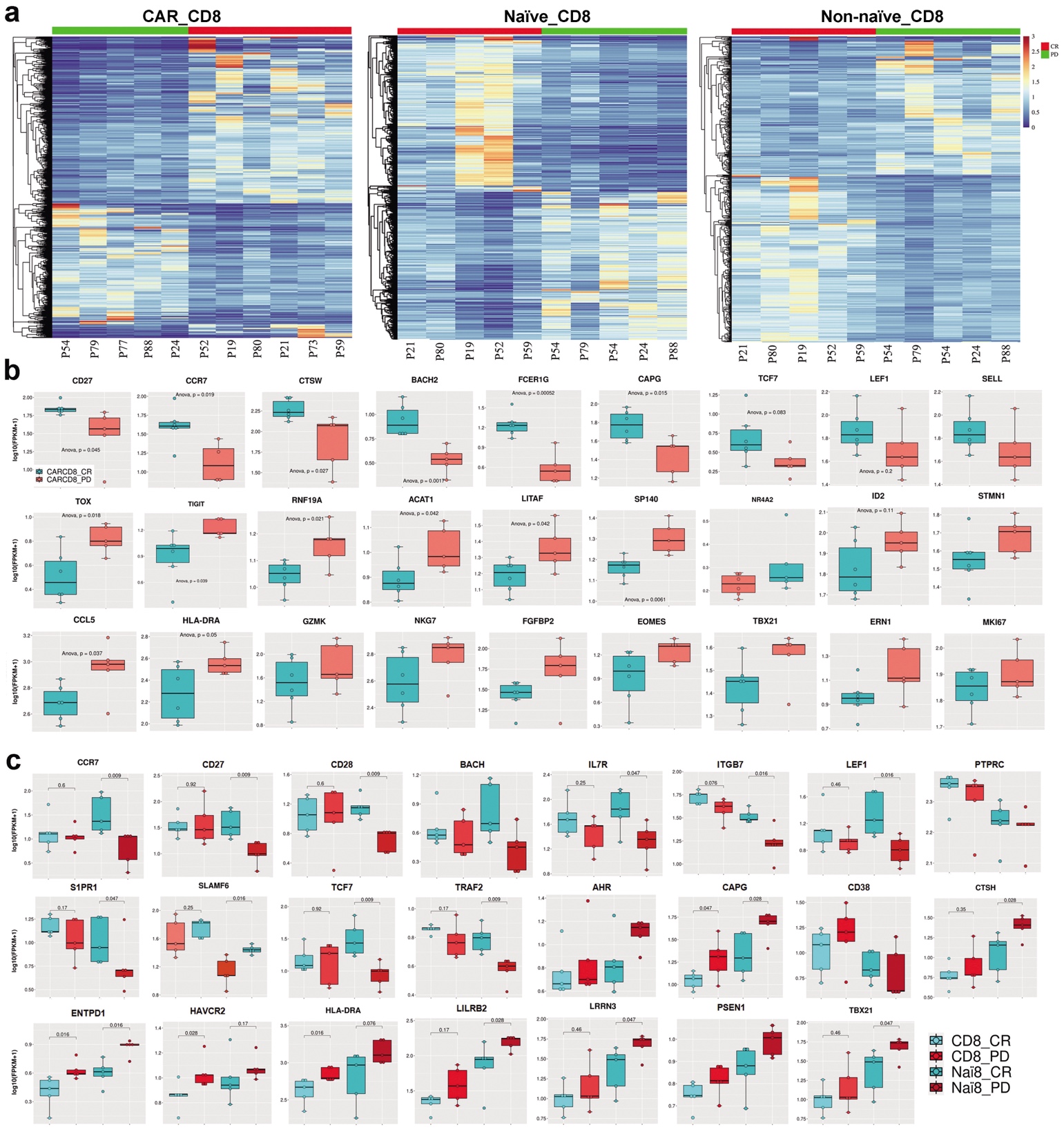


**Supplementary Figure. S4. The specified expressed genes** **between CD8^+^ T cells in DR patients and PD patients. a.** Hierarchical clustering of the RNA-seq analysis results shows differentially expressed genes between different cells of CR and PD patients. Box line diagram showing the specified expressed genes of between CD8^+^ CAR-T cells (**b**) and apheresis naïve CD8^+^ T cells and apheresis non-naïve CD8^+^ T cells (**c**) in CR patients and PD patients. Sequencing samples were sorted from 6 DR (P19, P21, P31, P59, P73, P80) and 6 resistant/PD (P24, P54, P56, P77, P79, P88) patients, CD8^+^ CAR-T cells from P56 patient, apheresis naïve CD8^+^ T cells and apheresis non-naïve CD8^+^ T cells from P73 patient, did not meet the criteria for sequencing.

**
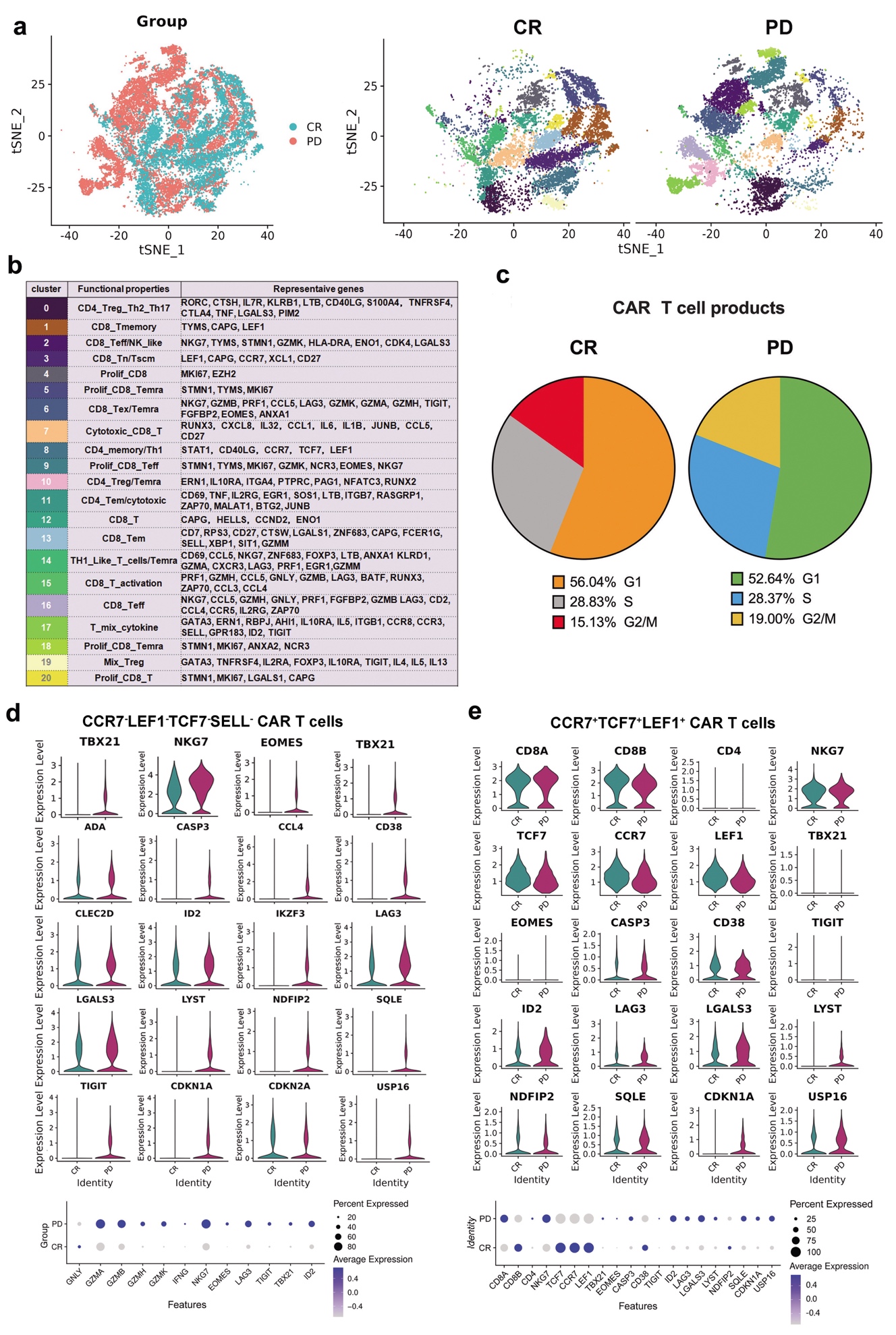
**

**Supplementary Figure. S5. Cellular characteristics of the differential expression of infused CAR-T cells from CR patients and PD patients. a.** Cells are color coded by CR and PD patients (**left**) and coded by clusters in CR and PD patients **(right)** in the tSNE plot. **b.** The function of different clusters was defined according to different expression genes. **c.** Sector graph showing the cell proportion with different cell cycles of infused CAR-T cells in CR patients and PD patients. Differences in gene expression between non-memory (CCR7^-^LEF1^-^TCF7^-^SELL^-^) cells **(d) and** CD8^+^CCR7^+^LEF1^+^TCF7^+^ cells **(e)** in infused CAR-T cells from CR patients and PD patients.


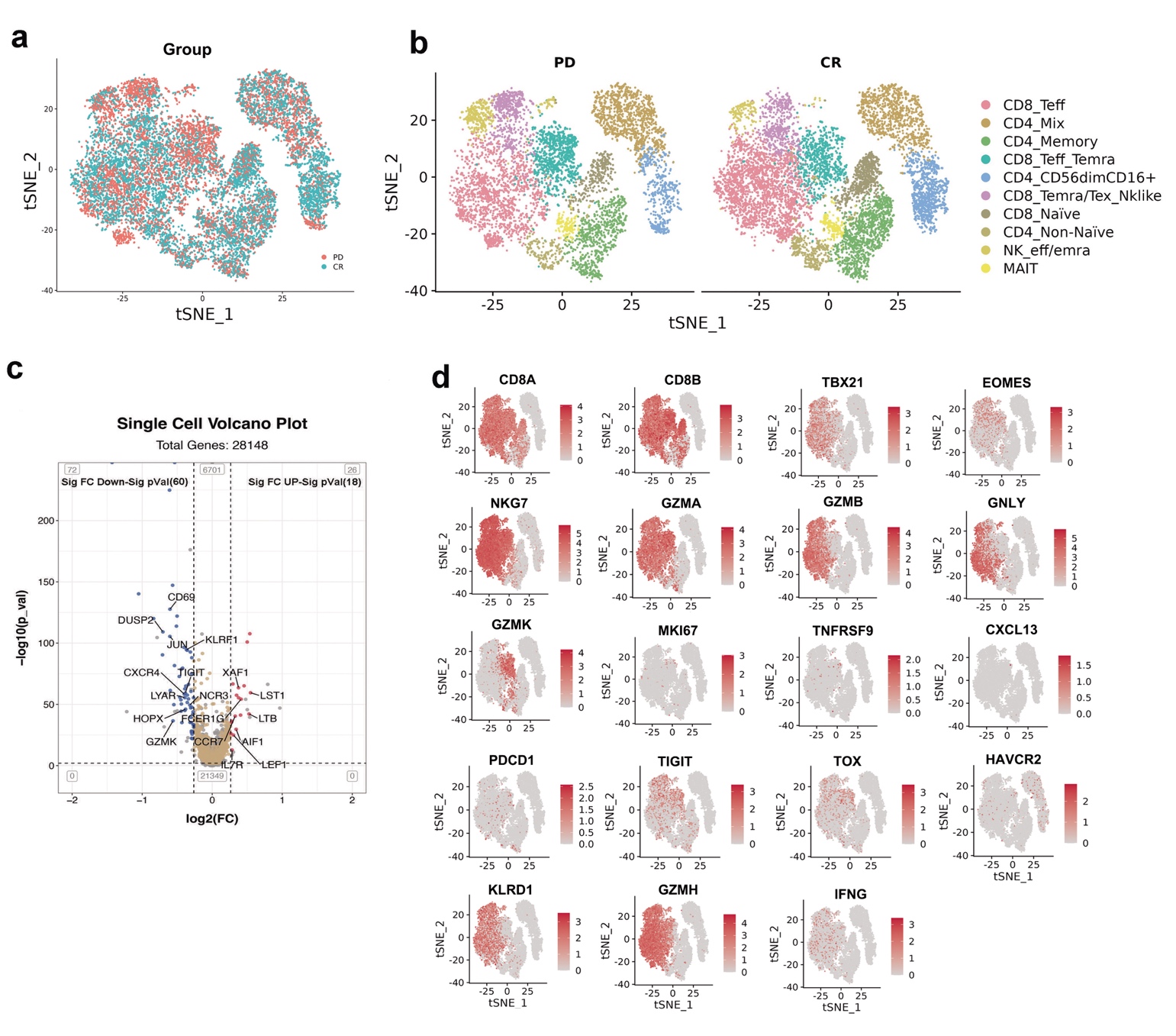


**Supplementary Figure. S6. Selected gene expression in apheresis T cell populations of all patients.** Cells are color coded by CR and PD patients (**a**) and coded by clusters in CR and PD patients **(b)** in the tSNE plot. **c.** Volcano plot showing differentially expressed genes comparing apheresis T cells in CR patients and PD patients. **d**. Expression of T cell activation/inhibitory related genes overlaid onto the tSNE plot.
